# Clinicohistopathological implications of MMP/VEGF expression in retinoblastoma: a combined meta-analysis and bioinformatics analysis

**DOI:** 10.1186/s12967-019-1975-3

**Published:** 2019-07-16

**Authors:** Jingyi Zhu, Xi Zhang, Liqianyu Ai, Rongdi Yuan, Jian Ye

**Affiliations:** 1 Department of Ophthalmology and Institute of Surgery Research, Daping Hospital, Army Medical University, Chongqing, 400042 China; 2 Department of Ophthalmology, Xinqiao Hospital, Army Medical University, Chongqing, 400042 China

**Keywords:** Matrix metalloproteinases, Retinoblastoma, Vascular endothelial growth factor, Bioinformatics analysis, Meta-analysis

## Abstract

**Background:**

No in-depth systematic evidence is available for assessing retinoblastoma malignancy and eligibility for subsequent treatment.

**Methods:**

The Cochrane Library, EMBASE, PubMed, Web of Science, and China Biology Medicine databases were searched, and 16 studies comprising 718 retinoblastoma patients were included. Pooled odds ratios (ORs) and summary correlation coefficients (r) with 95% confidence intervals (CIs) in random-effects, fixed-effects or quality-effects models were calculated using Review Manager 5.3 and MetaXL. GO functional annotation and KEGG pathway analysis were performed using the GO and STRING databases.

**Results:**

We observed significant associations between high levels of MMP-1 (OR, 4.21; 95% CI 1.86–9.54), MMP-2 (OR, 11.18; 95% CI 4.26–29.30), MMP-9 (OR, 10.41, 95% CI 4.26–25.47), and VEGF (OR, 8.09; 95% CI 4.03–16.20) with tumor invasion; high levels of MMP-1 (OR, 3.58; 95% CI 1.48–8.71), MMP-2 (OR, 2.96; 95% CI 1.32–6.64), MMP-9 (OR, 5.49; 95% CI 3.55–8.48) and VEGF (OR, 5.30; 95% CI 2.93–9.60) with poor differentiation; and overexpression of MMP-9 (OR, 5.17; 95% CI 2.85–9.38) with advanced clinical stages. Moreover, MMP-9 and VEGF expression were positively correlated (r, 0.61; 95% CI 0.38–0.77). Multiple GO terms were enriched associated with MMP-1, MMP-2, MMP-9 and VEGF, and they are closely associated with pathways, proteoglycans and microRNAs related to cancer.

**Conclusions:**

MMP-1, MMP-2, MMP-9 and VEGF play important roles in the development and progression of retinoblastoma. High levels of MMP-1, MMP-2, MMP-9 and VEGF are credible implications for increased malignancy, thus the need for more aggressive treatments.

**Electronic supplementary material:**

The online version of this article (10.1186/s12967-019-1975-3) contains supplementary material, which is available to authorized users.

## Background

Retinoblastoma is an extremely rare cancer that rapidly develops from immature retina cells, the light-detecting portion of the eye [1]. It is the most common pediatric malignant cancer of the eye and can easily lead to the loss of either sight or the eyeball and even death during early childhood [2]. The incidence and degree of malignancy of retinoblastoma are higher in developing countries than those in developed countries; moreover, cure rates remain unsatisfactory in developing countries, with relatively high morbidity and mortality, lower rates of eye salvage and higher rates of extraocular dissemination [3–5]. Survival rates in high-income countries can be greater than 95%, while the global survival rate is less than 30% [2]. Poor outcomes in retinoblastoma patients from developing countries are a concern worldwide.

The therapeutic options for retinoblastoma have undergone sweeping changes over the years; these mainly include chemotherapy, focal or consolidation therapy (e.g., laser photocoagulation, cryotherapy, thermotherapy and plaque brachytherapy), external beam radiotherapy and enucleation [6, 7]. While enucleation is progressively being supplanted by globe-sparing treatments to save the eyeball, this strategy may lead to compromised survival rates, as conservative treatments may not be as effective as enucleation for preventing tumor metastasis for potentially high-risk eyes. Therefore, the choice of conservative treatments with fewer side effects or aggressive therapies with greater survival rates is highly dependent on the assessment of the extent of intraocular disease and the extraocular tumor spread [7, 8]. However, in-depth systematic evidence available for estimating outcomes and eligibility for subsequent treatments is limited in retinoblastoma. Under these circumstances, it is crucial to identify biomarkers that predict retinoblastoma development, progression and clinical outcomes and can help determine the optimal treatment in the delicate balance between preserving the eyeball, vision and life.

Multiple markers have been suggested to be associated with the clinical features of retinoblastoma [9]. However, our preliminary search suggested that matrix metalloproteinases (MMPs) and vascular endothelial growth factor (VEGF) have been studied most. MMPs and VEGF regulate extracellular matrix (ECM) remodeling and microvascular permeability during angiogenesis [10–12]. The MMP family, a zinc-dependent endopeptidase family, regulates the degradation of the ECM and basal membrane [13]. Additionally, clinical studies have identified MMPs as prognostic markers and therapeutic targets in multiple types of cancer [14, 15]. VEGF is the most thoroughly and widely explored proangiogenic factor in tumors, including retinoblastoma, a heavily vascularized tumor [16–18]. Activated MMPs and VEGF are suggested to facilitate malignant cell growth, invasion, metastasis, angiogenesis and even chemoresistance in retinoblastoma [18–21]. The importance of these genes in retinoblastoma highlights their importance as candidates in our research. Moreover, MMPs and VEGF are closely related in oncogenesis and tumor progression, which increases our interest in their correlation [22–24]. For example, MMP-9 and MMP-2 inhibition may reduce VEGF expression and, thus, angiogenesis in retinoblastoma cell lines [25, 26]. However, whether MMPs and VEGF are coexpressed in retinoblastoma how they interact in pathways related to tumorigenesis and metastatic spread remain unknown.

Although remarkable progress in retinoblastoma therapies has been achieved, identification of systematic evidence for basing therapy recommendations or consensus has not progressed to the same extent. To the best of our knowledge, no systematic reviews or meta-analyses have investigated this issue. A scattering of single-center experimental studies exists, but the inadequate sample sizes of single studies reduce their credibility. Therefore, we conducted a meta-analysis to evaluate the significance of the MMPs/VEGF in predicting clinicopathological characteristics, thus providing comprehensive evidence for a better understanding of retinoblastoma biology, predicting prognosis and developing novel targeted therapies.

## Methods

### Search strategy

This meta-analysis was conducted following the Preferred Reporting Items for Systematic Reviews and Meta-Analyses (PRISMA) guidelines [27].

**Fig. 1:**
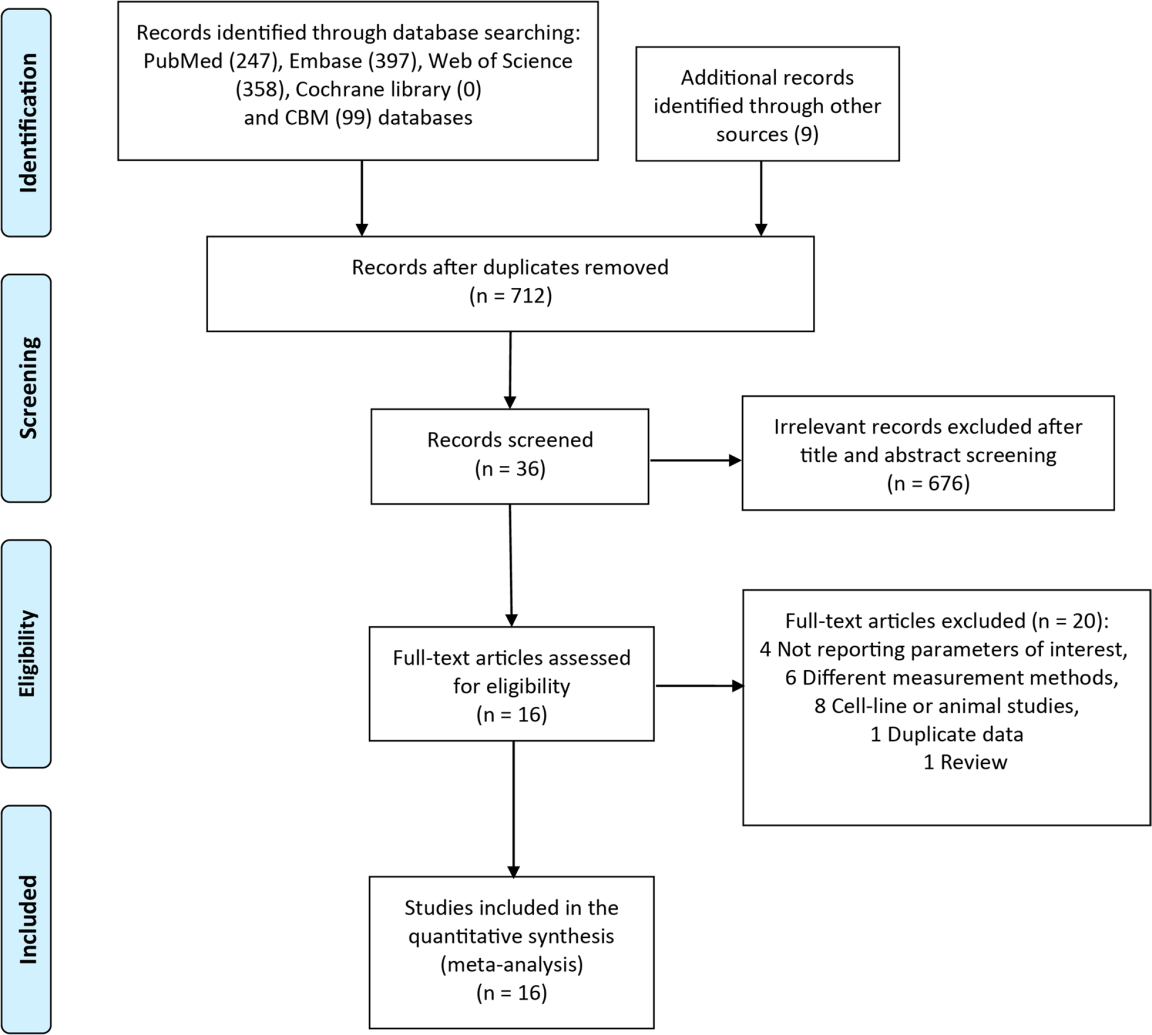
Diagram of the study selection process. This figure shows the diagram of study selection for the meta-analysis according PRISMA guidelines. *CBM* China Biology Medicine

A systematic search of the literature was conducted in PubMed, EMBASE, Cochran Library, and China Biology Medicine (CBM) through April 31, 2018. The following search strategy was applied: (“matrix metalloproteinase” or “MMP” or “stromelysin” or “collagenase” or “gelatinase” or “VEGF” or “vascular endothelial growth factor” or “VEGF-A”) and “retinoblastoma”. We retrieved 712 unique citations. Two authors examined the search results individually to identify relevant studies. Disagreements between the two reviewers were settled by discussion with a third reviewer. First, the title and abstract were screened to exclude obviously irrelevant articles. Then, full texts of articles were obtained and reviewed for eligibility. Reference lists of included articles and pertinent reviews were also searched (PRISMA flow diagram; Fig. 1).

### Eligibility criteria

Articles were included if the following inclusion criteria were met: (1) original clinical studies with clinical diagnosis of retinoblastoma; (2) studies that investigated associations between MMP or VEGF expression and clinicopathological parameters such as clinical stage, tumor differentiation and invasion; (3) protein expression analysis as measured by immunohistochemistry (IHC) assays; and (4) information for OR and r estimation that could be obtained directly from the article. The exclusion criteria were as follows: (1) case reports, reviews, editorial letters, comments, or nonhuman research; (2) duplicate publication in different journals or languages; and (3) studies in which patients underwent any presurgical treatment such as chemoreduction.

### Data extraction and quality assessment

Baseline demographic and clinical data were independently extracted by two investigators using a standard form and included the following information: author, year of publication, country of origin, patient baseline characteristics, detection method, cutoff value of positive expression and clinicopathological outcomes. After browsing all eligible trials, we selected MMP-1, MMP-2, MMP-9 and VEGF as our targeted molecules and tumor invasion, histodifferentiation and clinical stage as our outcomes. Other molecules and parameters were not evaluated due to insufficient data. During the data extraction, we noticed that many studies provide the coexpression pattern of candidate genes; therefore, we combined these data.

Study quality was assessed by the Newcastle–Ottawa Scale (NOS) with some modifications. The NOS is an 8-item scale for the assessment of study quality based on 3 aspects: patient selection, comparability and outcomes. The total score was 9 points, while a score ≥ 6 indicated good quality [28].

### Pathway analysis

Gene Ontology Analysis was conducted using the Functional Enrichment analysis tool (FunRich) [29, 30]. Results from the Kyoto Encyclopedia of Genes and Genomes (KEGG) were obtained through Cytoscape StringAPP [31], which can be accessed from the Search Tool for the Retrieval of Interacting Genes (STRING, https://string-db.org/) database [32]. GO analysis results with a Bonferroni corrected P value of less than 0.05 and KEGG pathways with a false discovery rate less than 0.05 were considered significant and displayed in the data.

### Integration of the PPI network

The interactive relationships among MMP-1, MMP-2, MMP-9, and VEGF were mapped using the online STRING database. Interactions with a combined score > 0.700 were defined as statistically significant.

### Statistical methods

Odds ratios (ORs) and their corresponding 95% confidence intervals (CIs) were calculated to assess the correlations between the candidate protein expression levels and clinicopathological parameters. If the corresponding 95% CI for each pooled OR did not overlap 1, then the summary effects were statistically significant.

Regarding the coexpression of MMP-9 and VEGF in retinoblastoma, outcomes were presented as r with 95% confidence intervals (CIs). Spearman correlation coefficients and sample size were extracted from the primary publications and combined by the statistical add-in software MetaXL (MetaXL, version 5.0; http://www.epigear.com/index_files/metaxl.html). Before weighting and pooling the data, Fisher’s z transformation was performed to stabilize the variance of the r, which would otherwise tend to grow smaller as r approaches 1. Detailed information for Fisher’s z transformation is included in the “MetaXL User Guide”. If the pooled r was larger than 0, and the corresponding 95% CI did not overlap 0, the two genes were considered coexpressed [33].

Statistical heterogeneity was estimated using a Chi square test and a Q-test. Quality-effects (QE), random-effects (RE) and fixed-effects (FE) models were all used in the analyses to determine whether the conclusion was consistent and stable. However, when I^2^ ≥ 50% or the P value for the Q-test ≤ 0.10, heterogeneity was considered to be significant, in which case the FE model for summary estimation was abandoned. For convenience, the OR in this paper refers to the RE model without specific comments.

Sensitivity analysis was conducted using several methods to verify the validity and reliability of the conclusions. Each individual study was removed successively to ascertain its effects on the results. In addition, different models (RE, FE and QE) were applied using Review Manager version 5.3 and MetaXL to confirm the stability of the results. The QE model is a new estimation method implemented in MetaXL. It is a modified version of the FE model that does not consider the level of heterogeneity and represents a more convincing alternative to the RE model [34, 35].

We used Begg’s funnel plots to evaluate the publication bias of included studies. However, accurate appraisal was not possible due to the limited number of studies.

## Results

### Inclusion of studies

A diagram of the literature search is shown in Fig. 1. A total of 712 records published before April 31, 2018, were identified through searches of the online databases. After screening the titles and abstracts and reviewing the full text, a large number of articles (676 references) were discarded, mainly because most studies are irrelevant to retinoblastoma (Fig. 1). Finally, 16 primary articles with 718 patients (725 eyes) conducted in China, India and Egypt that satisfied the inclusion criteria were included [9, 36–50]. For patients with bilateral retinoblastoma, we randomly selected one eye and pooled the effects with the data provided. When the requisite information was not available, both eyes were included.

Table 1 shows an overview of the included studies. The studies were confined to China, Egypt and India, likely because these populous countries encompass more retinoblastoma patients [2, 51]. Additionally, the outcomes of retinoblastoma patients are still unsatisfactory in these developing countries [2, 4]. The main results of the meta-analysis are summarized in Table 2.

**Table 1:** Main characteristics of studies included in the meta-analysis

Study	Country	Inclusion period	Number of patients	Gender	Eye	Age	Clinical staging	Tumor invasion	Histological type	Preoperative treatment	Detection method	Expression evaluation standards	MMP types	NOS score
				Male/female	Right/left		/II+III	N_0_/N_X_	Well + moderately/poorly diff					
Yun Li et al. (2006)	China	1996–2005	30	18/12	NA	Range 4 month–6 years Median 2.9 years	13/17	17/13	11/19	NA	IHC	Proportion	MMP-1 MMP-9	8
Haiyan Li et al. (2012)	China	2002–2011	52	31/21	33/29	Range 6 month–9 years Mean 5.1 ± 1.2 years	NA	28/24	24/28	No chemo	IHC	Proportion + intensity	MMP-1 MMP-9 VEGF	7
Siqi Yuan et al. (2010)	China	2000–2009	31	23/18	NA	Range 5 month–9 years Median 3.3 years	NA	17/14	13/18	NA	IHC	Proportion + intensity	MMP-1 VEGF	6
Mohan Adithi et al. (2006)	India	2000–2003	55	35/20	29/26	Range 1 month–14 years Median 2 years	0/55	23/32	21/34	No chemo	IHC	Proportion	MMP-2 MMP-9	8
Yang Yang et al. (2013)	China	2001–2012	45	23/22	24/21	Range 2 month–8 years	36/9	26/19	17/28	No chemo No radio	IHC	Proportion	MMP-2	6
Lin Zhou et al. (2010)	China	1989–2010	40	23/17	17/23	Range 4 month–18 years Median 2.9 years	NA	27/13	15/25	No chemo No radio	IHC	Proportion + intensity	MMP-2 MMP-9 VEGF	6
Jun Liang et al. (2017)	China	2011–2016	100	66/34	NA	Range 4 month–11 years Median 4.2 years	39/61	NA	55/45	NA	IHC	Proportion + intensity	MMP-9 VEGF	8
Yan Sun et al. (2014)	China	2000–2011	56	29/27	NA	Range 3 month–7 years Mean 3.6 years	20/16	28/28	31/25	No chemo	IHC	Proportion + intensity	MMP-9	7
Zhen Ge et al. (2007)	China	1995–2005	32	19/13	NA	Range 4 month–10 years Median 3.1 years	NA	21/11	12/20	No chemo	IHC	Proportion + intensity	MMP-9 VEGF	6
Zixu Wu et al. (2011)	China	2000–2010	41	23/18	NA	NA	29/12	24/17	20/21	NA	IHC	Proportion + intensity	MMP-9	6
Jia Yu et al. (2009)	China	1990–2006	47	25/12	20/27	Range 6 month–10 years	NA	32/15	17/30	No chemo No radio	IHC	Proportion + intensity	MMP-9 VEGF	6
Yuejun Liu et al. (2014)	China	2010–2014	30	18/12	NA	Mean 5.2 years	NA	17/13	12/18	No chemo	IHC	Proportion + intensity	MMP-9	6
Ying Jiang et al. (2004)	China	1999–2002	22	NA	NA	Range 2 month–5 years Mean 2.4 years	NA	14/8	8/14	No chemo No radio	IHC	Proportion	VEGF	7
Nermeen S Youssef et al. (2014)	Egypt	2009–2013	56	24/32	23/40	Mean 20.94 m ± 11.75 months	NA	26/30	36/20	No preoperative adjunctive treatments	IHC	percentage	VEGF	8
Lijuan Meng et al. (2011)	China	NA	48	27/21	22/26	Range 5 month–8y	NA	21/27	19/29	No chemo No radio	IHC	Proportion × intensity	MMP-9 VEGF	6
Li Fang et al. (2010)	China	2000–2007	33	18/15	14/19	Range 6 month–9 years	NA	22/11	12/21	No chemo No radio	IHC	Proportion + intensity	VEGF	6

*m* month, *y* year, *IHC* immunohistochemistry, *NA* not available, *chemo* chemotherapy, *radi* radiation therapy, *diff* differentiation, *N0*: absent local invasion, *Nx* optic nerve, optic nerve, choroidal, or scleral invasion, *Proportion* percentage of positive cells, *intensity* staining intensity of positive cells

**Table 2:** Summary of the main findings from the meta-analysis

Outcome-molecular	No. of patients/studies	Random-effects model			Quality-effects model^a^		Fixed-effects model^b^		
		OR(s) or r (95% CI)	Test for overall effects (P)	Overall heterogeneity (I^2^), %	OR(s) or r (95% CI)	Overall Heterogeneity (I^2^), %	OR(s) or r (95% CI)	Test for overall effects (P)	Overall heterogeneity (I^2^), %
Tumor invasion		OR			OR		OR		
MMP-1	113/3	4.21 (1.86–9.54)	0.0006	0	4.14 (1.83–9.39)	0	4.21 (1.87.9/47)	0.0005	0
MMP-2	140/3	11.18 (4.26–29.30)	< 0.00001	0	11.24 (4.29–29.50)	0	11.46 (4.41–29.79)	< 0.00001	0
MMP-9	431/10	10.41 (4.26–25.47)	< 0.00001	59	6.37 (2.48–16.35)	57	–	–	–
VEGF	409/10	8.09 (4.03–16.20)	< 0.00001	0	8.08 (4.03–16.19)	0	9.93 (5.11–19.30)	< 0.00001	0
Tumor differentiation		OR			OR		OR		
MMP-1	113/3	3.58 (1.48–8.71)	0.005	13	3.42 (1.40–8.33)	13	3.55 (1.60–7.89)	0.002	13
MMP-2	140/3	2.96 (1.32–6.64)	0.009	13	3.03 (1.35–6.81)	13	3.01 (1.45–6.25)	0.003	13
MMP-9	474/10	5.49 (3.55–8.48)	< 0.00001	0	6.37 (2.48–16.35)	57	5.77 (3.77–8.83)	< 0.00001	0
VEGF	510/11	5.30 (2.93–9.60)	< 0.00001	38	4.93 (2.71–8.96)	0	5.04 (3.23–7.87)	< 0.00001	38
Clinical stage		OR			OR		OR		
MMP-9	227/4	5.17 (2.85–9.38)	< 0.00001	0	5.22 (2.88–9.48)	0	5.19 (2.87–9.39)	< 0.00001	0
Gender		OR			OR		OR		
MMP-9	152/3	0.60 (0.26–1.34)	0.21	32	0.60 (0.27–1.36)	32	0.60 (0.31–1.15)	0.12	32
Coexpression^a^		r			r				
MMP-9 and VEGF	287/5	0.61 (0.38–0.77)	–	84	0.59 (0.34–0.77)	84	–	–	–

*No* number, *OR* odds ratio, *r* summary correlation coefficients, *CI* confidence intervals

^a^These results are calculated using statistical add-in software MetaXL, in which the p value of testing for overall effects is not provided

^b^The fixed-effects model is applied only when I^2^ ≤ 50%

### Expression and clinicopathological characteristics

Three members of the MMP family, MMP-1, MMP-2 and MMP-9, were assessed after computer and manual searches. Among the remaining members, only MMP-14 was reported in two related studies, which was still insufficient to conduct a meta-analysis. Therefore, the remaining members of the MMP family are not included. Data on the following tumor-related parameters were extracted: histological differentiation, clinical stage and tumor invasion of the retinoblastoma. Specifically, tumor invasion data in most of the included studies were related to optic nerve invasion, except Mohan’s report, which included the choroid, optic nerve and orbit [41, 52]. In our analysis, tumor invasion is divided into two classes; any kinds of optic nerve invasion detected is defined as N_X_ (pre/post laminar, cut end, etc.), otherwise it is N_0_. The retinoblastoma stages in enrolled trials differed,thus we reclassified them into two stages: Localized/Regional stage and advanced stage. Localized/Regional stage refers to stage (I + II), and advanced tumor stage is defined as stage (III + IV) in International Classification of Intraocular Retinoblastoma (IIRC) developed by Murphree [53].

Table 2 shows major conclusions from our meta-analysis.

**Fig. 2:**
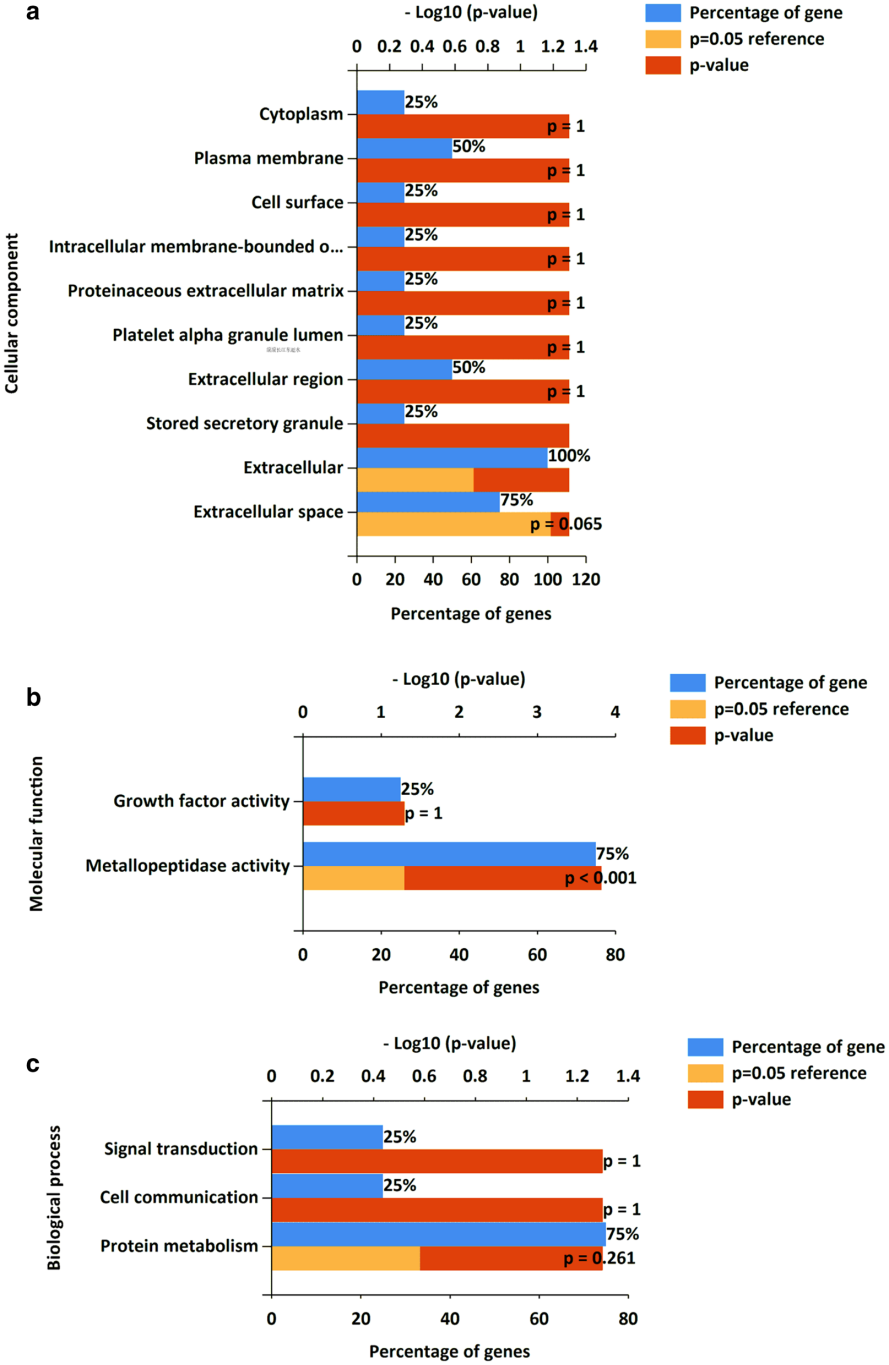
Gene ontology enrichment analysis for MMP-1, MMP-2, MMP-9 and VEGF. This figure presents a representative, partial list of the significantly enriched GO terms associated with MMP-1, MMP-2, MMP-9 and VEGF in the cellular component (**a**), molecular function (**b**) and biological process (**c**) categories

We revealed that increased expression of MMP-1 (OR = 4.21, 95% CI 1.86–9.54, P = 0.0006, RE model; OR = 4.14, 95% CI 1.83–9.39, QE model), MMP-2 (OR = 11.18, 95% CI 4.26–29.30, P < 0.00001, RE model; OR = 11.24, 95% CI 4.29–29.50, QE model), MMP-9 (OR = 10.41, 95% CI 4.26–25.47, P < 0.00001, RE model; OR = 6.37, 95% CI 2.48–16.35, QE model), and VEGF (OR = 8.09, 95% CI 4.03–16.20, P < 0.00001, RE model; OR = 8.08, 95% CI 4.03–16.19, QE model) was significantly associated with tumor metastasis (Fig. 2). No heterogeneity was found (MMP-1: P = 0.61, I^2^ = 0%; MMP-2: P = 0.70, I^2^ = 0%; VEGF: P = 0.55, I^2^ = 0%), except for MMP-9 (P = 0.009, I^2^ = 59%) (Additional file 1: Figure S1). An FE model was also applied, and the conclusions are consistent with results from the RE model (data not shown).

The association of gene expression level with tumor differentiation was also investigated. Overexpression of MMP-1 (OR = 3.58, 95% CI 1.48–8.71, P = 0.005, RE model; OR = 3.42, 95% CI 1.40–8.33, QE model), MMP-2 (OR = 2.96, 95% CI 1.32–6.64, P = 0.009, RE model; OR = 3.03, 95% CI 1.35–6.81, QE model), MMP-9 (OR = 5.49, 95% CI 3.55–8.48, P < 0.00001, RE model; OR = 5.50, 95% CI 3.55–8.51, QE model) and VEGF (OR = 5.30, 95% CI 2.93–9.60, P < 0.00001, RE model; OR = 4.93, 95% CI 2.71–8.96, QE model) was related to poor histological differentiation (Additional file 2: Figure S2). No obvious heterogeneity was identified among all involved subjects (MMP-1: P = 0.32, I^2^ = 13%; MMP-2: P = 0.32, I^2^ = 13%; MMP-9: P = 0.74, I^2^ = 0%; VEGF: P = 0.10, I^2^ = 38%) (Additional file 2: Figure S2). Similar results were attained using the FE model (data not shown).

We failed to study the relationship between these molecules and other clinicopathological parameters of retinoblastoma due to insufficient original data. However, we determined that higher MMP-9 levels correlate with advanced tumor stage (OR = 5.17, 95% CI 2.85–9.38, P < 0.00001, RE model; OR = 5.22, 95% CI 2.88–9.48, P < 0.00001, QE model) (Additional file 3: Figure S3), independent of gender (OR = 0.60, 95% CI 0.26–1.34, P = 0.21, RE model; OR = 0.60, 95% CI 0.27–1.36, P = 0.12, QE model) (Additional file 3: Figure S3). Pooled data from the FE model led to the same conclusion (data not shown). No significant heterogeneity was observed among these studies (tumor stage: P = 0.83, I^2^ = 0%; gender: P = 0.23, I^2^ = 32%) (Additional file 3: Figure S3).

### Coexpression of MMP-9 and VEGF

VEGF and MMPs, especially MMP-9, often work in parallel in various pathological conditions. Using STRING (a database covering 9,643,763 proteins from 2031 organisms), we constructed a PPI, which showed the relationship between MMPs and VEGF. There were 4 nodes and 6 edges in the network, confirming that VEGF is correlated with all three other proteins (Additional file 4: Figure S4). Next, we investigated MMP-9 and VEGF coexpression in retinoblastoma tissue, and demonstrated that MMP-9 and VEGF expression are positively correlated (r = 0.61, 95% CI 0.38–0.77, RE model; r = 0.59, 95% CI 0.34–0.77, QE model) (Additional file 3: Figure S3C). However, the studies exhibited significant heterogeneity (P = 0.00, I^2^ = 84% for both models). Therefore, the FE model was not used.

### Bioinformatics analysis of MMP-1, MMP-2, MMP-9 and VEGF

To determine the functions of MMP-1, MMP-2, MMP-9 and VEGF in retinoblastoma, these four genes were mapped to the Gene Ontology (GO) database. The GO terms including biological process (BP), cellular component (CC) and molecular function (MF) were performed and shown in Fig. 2 based on the detection P value. These four genes are associated to functions enriched in multiple components, such as cytoplasm, cell surface, plasma membrane and extracellular region. In the BP term, process including signal transduction, cell communication and protein metabolism were enriched; the functions were predominantly associated with growth factor activity and metalloproteinases activity. The KEGG pathway is shown in Table 3, which confirmed that these four genes are closely related to cancer and the estrogen signaling pathway.

**Table 3:** KEGG pathway

Pathway description	Observed gene count	False discovery rate	Matching proteins
Pathways in cancer	4	8.43E−06	MMP1, MMP2, MMP9, VEGFA
Proteoglycans in cancer	3	0.000453	MMP1, MMP2, MMP9, VEGFA
Estrogen signaling pathway	2	0.007	MMP2, MMP9, VEGFA
Leukocyte transendothelial migration	2	0.00862	MMP1, VEGFA
MicroRNAs in cancer	2	0.0118	MMP9, VEGFA

### Assessments of potential biases and sensitivity

Sensitivity analyses were conducted by sequentially omitting each study and repeating the analysis (Additional file 5: Figure S5). No individual study was found to contribute effects that were able to change the overall trends; therefore, the results of the meta-analysis are stable. Findings from different models further supported our results.

Publication bias was evaluated by visual inspection of Begg’s funnel plot. Asymmetry was noted in the analysis of the association between tumor invasion and VEGF expression, as well as the correlation between MMP-9 and VEGF expression in retinoblastoma, indicating possible publication bias (Additional file 6: Figure S6).

## Discussion

Retinoblastoma is a curable intraocular tumor of the pediatric population [1, 54]. However, due to relatively late detection and primitive medical care, retinoblastoma appears to be a serious vision- and life-threatening disease in developing countries. Management of retinoblastoma depends on cooperative, multidisciplinary efforts and comprehensive surveillance and evaluation of the tumor [6, 55]. Unfortunately, we have lagged behind in providing systematic evidence on which to base the choice of treatment; nevertheless, great progress has been achieved in therapeutic methods for retinoblastoma. Thus, our systematic review and meta-analysis sought to contribute missing data examining tumor invasion, differentiation and clinical stages in the hope of more precisely predicting the long-term outcomes and determining individualized therapies for retinoblastoma.

In this meta-analysis, we assessed the clinicopathological significance of MMPs and VEGF in retinoblastoma and detected a potential relationship between MMP-9 and VEGF expression. The results confirm that MMP-1, MMP-2, MMP-9, and VEGF overexpression is highly related to poor retinoblastoma differentiation and tumor invasion. Moreover, MMP-9 overexpression is associated with tumor stage, regardless of the gender of the patients. Except for MMP-1, the lower confidence intervals (LCIs) of the ORs are all greater than 3, which implied intensely significant correlations. The findings suggest that these genes participate in tumorigenesis and metastasis, which is in agreement with previous experimental studies. However, according to the study by Nermeen, overexpression of VEGF may be irrelevant to poor differentiation [9], whereas our study of a Chinese population reported the opposite result. This contradiction implies that VEGF may play distinct roles in retinoblastoma oncogenesis among different races.

Multiple measures, including quality assessment, public bias evaluation, and sensitivity and heterogeneity analyses have been adopted to control potential biases while exploring the main outcomes. The quality of studies included in this analysis was acceptable, as their NOS scores were all greater than 6. Regarding publication bias, the funnel plots display asymmetry between certain groups, probably due to language and ethic confinement in enrolled studies. Additionally, it is possible that MMP/VEGF expression in progressed retinoblastoma has been previously reported in other studies, leading more researchers to validate the findings in their specific patient populations. The limited number of trials deters us from further investigation. Aside from conventional meta-analysis, our study employs a novel QE model in addition to the FE and RE models to pool the effects. The QE model is an updated estimator for meta-analyses and has the advantage of a decreased mean squared error and reduced observed variance compared with the RE model. Regardless of the level of heterogeneity, the QE model maintains the correct coverage probability of the confidence interval [56]. With three different models to weight the average, our results should be more convincing. Certain included studies introduced a high level of heterogeneity across studies, such as Nermeen’s study as shown in Additional file 2: Figure S2D, Mohan’s study as shown in Additional file 3: Figure S3B and Lin Zhou’s study as shown in Additional file 3: Figure S3C, but their removal did not lead to changed conclusions, implying that the results are relatively stable.

Although prognostic markers are widely investigated and applied in other malignant tumors, such as lung cancer, they have remained undetermined in retinoblastoma [57, 58]. Possible reasons include an insufficient number of samples due to the rarity of the disease and cautious application of biopsy. Fearing tumor dissemination and extraocular metastasis development, the diagnosis and grading of retinoblastoma using biopsy are usually discouraged [59]. However, several biopsy techniques, such as fine needle aspiration cytology and lipid biopsy (using the aqueous humor as a surrogate biopsy material for retinoblastoma DNA), have emerged as safe and effective methods [59, 60]. Nevertheless, long-term follow-up in a larger cohort of patients is needed to validate the results and provide safety data. To the best of our knowledge, our paper is the first meta-analysis to provide a series of markers for predicting retinoblastoma prognosis. These oncogenes contribute to a more accurate estimation of clinical outcomes and provide early indications for possible retinoblastoma metastasis. Patients with high protein expression are considered to be at high-risk and potential candidates for aggressive treatment. In addition, these proteins may help monitor patients’ responses to therapy and facilitate further treatments.

Major advances have been made in small molecule therapeutics. Targets such as inhibitors of the MDMX-p53 response, histone deacetylase inhibitors, and spleen tyrosine kinase inhibitors have shown promise in the treatment of retinoblastoma, with minimal off-target effects in animal models or clinic trials [61, 62]. Our study supplements MMPs and VEGF as promising druggable molecular targets for retinoblastoma treatment. Retinoblastoma exhibits invasive behavior to adjacent tissues and the blood stream at the early stage of the tumor, which emphasizes the urgency of preventing retinoblastoma metastasis at the early stage [4, 63]. However, although current therapy focuses on blocking tumor cell division and tumor growth, specific treatments targeted to prevent retinoblastoma metastasis and invasion are unavailable. Our results may pioneer the first indication for targets that interfere with tumor spread. Additionally, molecular suppression or silencing of these targets has the advantage of superior selectivity and lower systemic toxicities compared to chemotherapeutic agents. The use of an anti-VEGF antibody in combination with chemotherapy may enhance the efficacy of chemotherapy toward retinoblastoma [64, 65], despite a distinct toxicity profile in use for clinical trials. Whether a VEGF inhibitor will work effectively in humans and not only in nonhuman or human tumor sample trials needs to be validated as well.

We have demonstrated prognostic concordance among targeted gene expression signatures. Next, we investigated how the various genes are related to one another. Therefore, we further investigated the associations among gene targets. Regrettably, due to a lack of original studies, we were only able to show that MMP-9 and VEGF are coexpressed. Nonetheless, this represents a novel piece of system-level evidence elucidating the cellular pathways and biological processes regulating retinoblastoma phenotype, progression and prognostic performance. The identification of a synchronized overexpression pattern between MMP-9 and VEGF may generate many insights into tumor biology and pathogenesis. Notably, the degree of overlap among regulatory programs greatly influences the degree of coexpression [66]. According to previous studies, MMP-9 and VEGF are indeed closely related on the cellular level. MMP-9 plays an essential role in the acquisition of an angiogenic phenotype by assisting in VEGF liberation from the ECM and is involved in VEGF–VEGF-receptor interactions [23, 67, 68]. VEGF can also induce MMP-9 expression through pathways such as the Ras-activated extracellular signal-regulated kinase (ERK) pathway [24, 69]. In addition to positive feedback regulation, MMP-9 and VEGF also share a generous number of upstream pathways. Although we confirmed that MMP-9 and VEGF are coexpressed in retinoblastoma, further exploration is needed to identify the biological role of their interaction.

Last but not least, the GO functional annotation and KEGG pathway analysis of the four target genes provides additional information about their roles in retinoblastoma, indicating that they play important roles in multiple cellular components, thus promoting tumorigenesis and metastasis, which prompt potential directions for further investigations.

## Limitations

This study has some inherent limitations that should be highlighted. First, although all studies utilized IHC, the antibodies used are different, and the threshold value is inconsistent among trials. Thus, despite the implementation of a standard regimen to avoid divergence, heterogeneity from these factors is inevitable. Ethnic groups also vary, but subgroup analysis of different ethnic groups is not feasible due to the limited number of trails. Second, prognostic indexes, such as overall survival and disease-free survival, were not end points for most included studies, which restricts our study to an analysis of clinicohistopathological characteristics. Life-long follow-up should be performed in subsequent studies, as retinoblastoma survivors are at high risk of developing secondary cancers [70, 71]. Third, our search was restricted to studies published in English or Chinese, which may be responsible for the publication bias. Our results are more applicable to certain ethnic groups; as most relevant reports were conducted in Chinese populations. Outcomes in developing countries are relatively unsatisfactory [2], so these studies may add additional bias. Fourth, tumor invasion data in most of the included studies were related to optic nerve invasion except Mohan’s report, which included the choroid, optic nerve and orbit [41]. Optic nerve invasion is emphasized mainly because it is the most common route for retinoblastoma to progress into the brain and then further metastasize [52]. However, excluding other types of invasion may reduce the available information in the early detection of retinoblastoma progression. Finally, analyses of MMP-1 and MMP-2 expression are based on only 3 studies. Corresponding results based on small samples should be interpreted with caution.

## Conclusions

We concluded that the overexpression of MMP-1, MMP-2, MMP-9 and VEGF is highly related to poor retinoblastoma differentiation, tumor invasion and advanced clinical stage and, thus, have a role in predicting the prognosis of retinoblastoma patients. Meanwhile, MMP-9 and VEGF proteins exhibit a system-level coexpression pattern in retinoblastoma, indicating their potentially important biological relationships in tumor development and progression.

## Additional files

**Additional file 1: Figure S1.** Association between MMP/VEGF expression and retinoblastoma invasion. (A) MMP-1. (B) MMP-2. (C) MMP-9. (D) VEGF. The forest plots on the left side show the results of the random-effects model generated using Review Manager. The forest plots on the right side were generated using MetaXL with a quality-effects model.

**Additional file 2: Figure S2.** Association between MMP/VEGF expression and retinoblastoma differentiation. (A) MMP-1. (B) MMP-2. (C) MMP-9. (D) VEGF. The forest plots on the left side show the results of the random-effects model generated using Review Manager. The forest plots on the right side were generated using MetaXL with a quality-effects model.

**Additional file 3: Figure S3.** Association between MMP-9 expression and retinoblastoma clinical stage, patient gender, and VEGF expression. (A) MMP-9 and clinical stage. (B) MMP-9 and patient gender. (C) MMP-9 and VEGF coexpression. The forest plots on the left side show the results of the random-effects model generated using Review Manager. The forest plots on the right side were generated using MetaXL with a quality-effects model.

**Additional file 4: Figure S4.** Protein–protein interaction (PPI) networks between MMP-1, MMP-2, MMP-9 and VEGF. Edges with different colors represent protein–protein associations. Blue edges represent the association from curated databases. Yellow edges have confirmed association by text mining. Purple edges represent the protein homology.

**Additional file 5: Figure S5.** Sensitivity analysis evaluating the impact of individual studies on the pooled results. (A) MMP-1 and invasion. (B) MMP-1 and differentiation. (C) MMP-2 and invasion. (D) MMP-2 and differentiation. (E) MMP-9 and invasion. (F) MMP-9 and differentiation. (G) MMP-9 and stage. (H) MMP-9 and gender. (I) VEGF and invasion. (J) VEGF and differentiation. (K) MMP-9 and VEGF coexpression.

**Additional file 6: Figure S6.** Funnel plot for publication bias. (A) Tumor invasion. (B) Tumor differentiation. (C) Clinical stage. (D) Gender. (E) Coexpression of MMP-9 and VEGF.

## Data Availability

All the data and materials supporting the conclusions are included in the main paper.

## Abbreviations

MMP: matrix metalloproteinase
VEGF: vascular endothelial growth factor
PRISMA: Preferred Reporting Items for Systematic Reviews and Meta-Analyses
CBM: China Biology Medicine
LCIs: lower confidence intervals
ORs: odds ratios
BP: biological process
CC: cellular component
MF: molecular function
GO: gene ontology
KEGG: Kyoto Encyclopedia of Genes and Genomes
IHC: immunohistochemistry
