# Supplementary figures and images for: Clinicohistopathological implications of MMP/VEGF expression in retinoblastoma: a combined meta-analysis and bioinformatics analysis
Source: J Transl Med. 2019 Jul 16;17:226. doi: 10.1186/s12967-019-1975-3 (PMC6636009; doi:10.1186/s12967-019-1975-3)

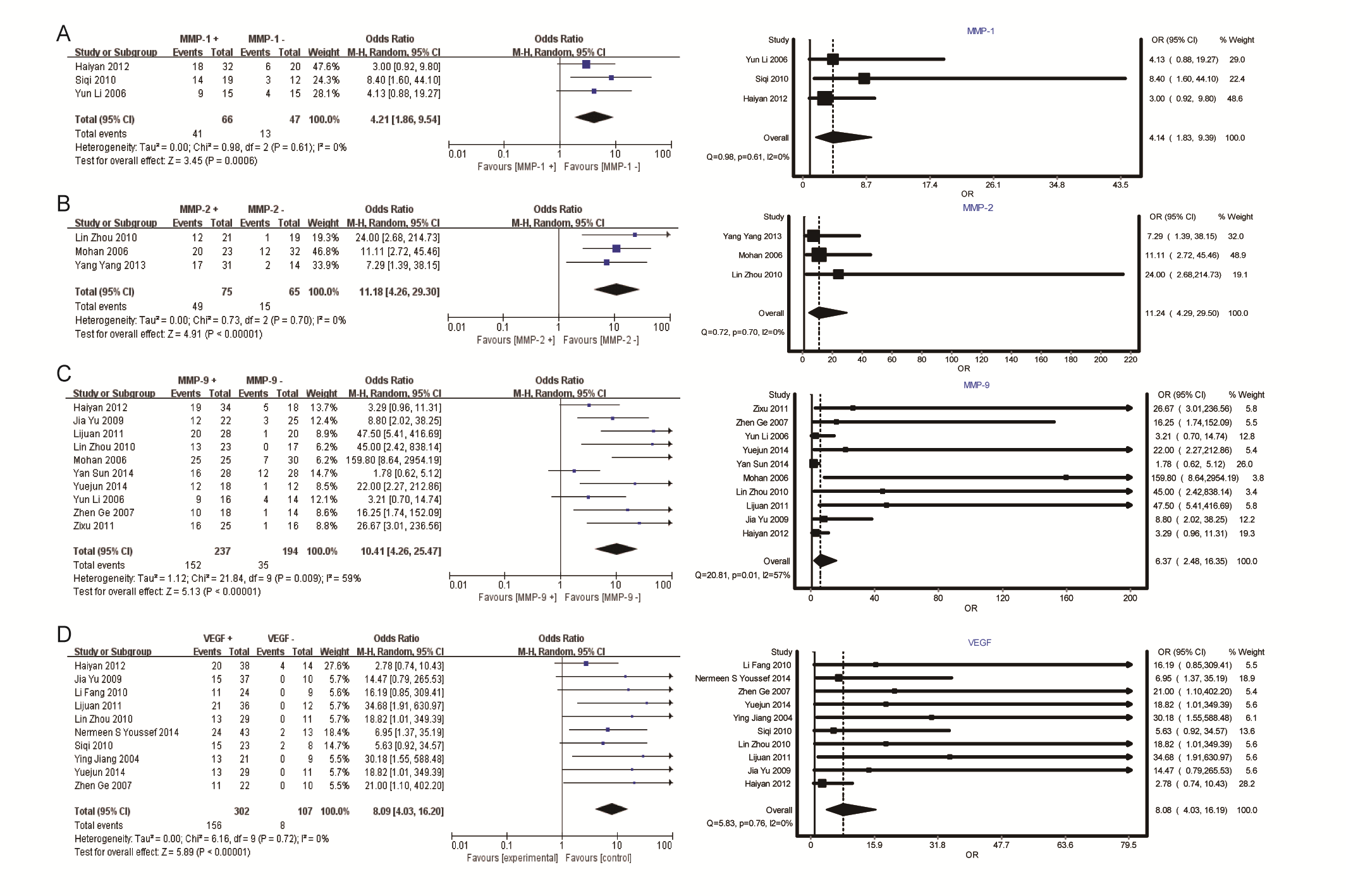

Supplement: Supplementary file 1 — Additional file 1: Figure S1. Association between MMP/VEGF expression and retinoblastoma invasion. (A) MMP-1. (B) MMP-2. (C) MMP-9. (D) VEGF. The forest plots on the left side show the results of the random-effects model generated using Review Manager. The forest plots on the right side were generated using MetaXL with a quality-effects model. [file 12967_2019_1975_MOESM1_ESM.png]

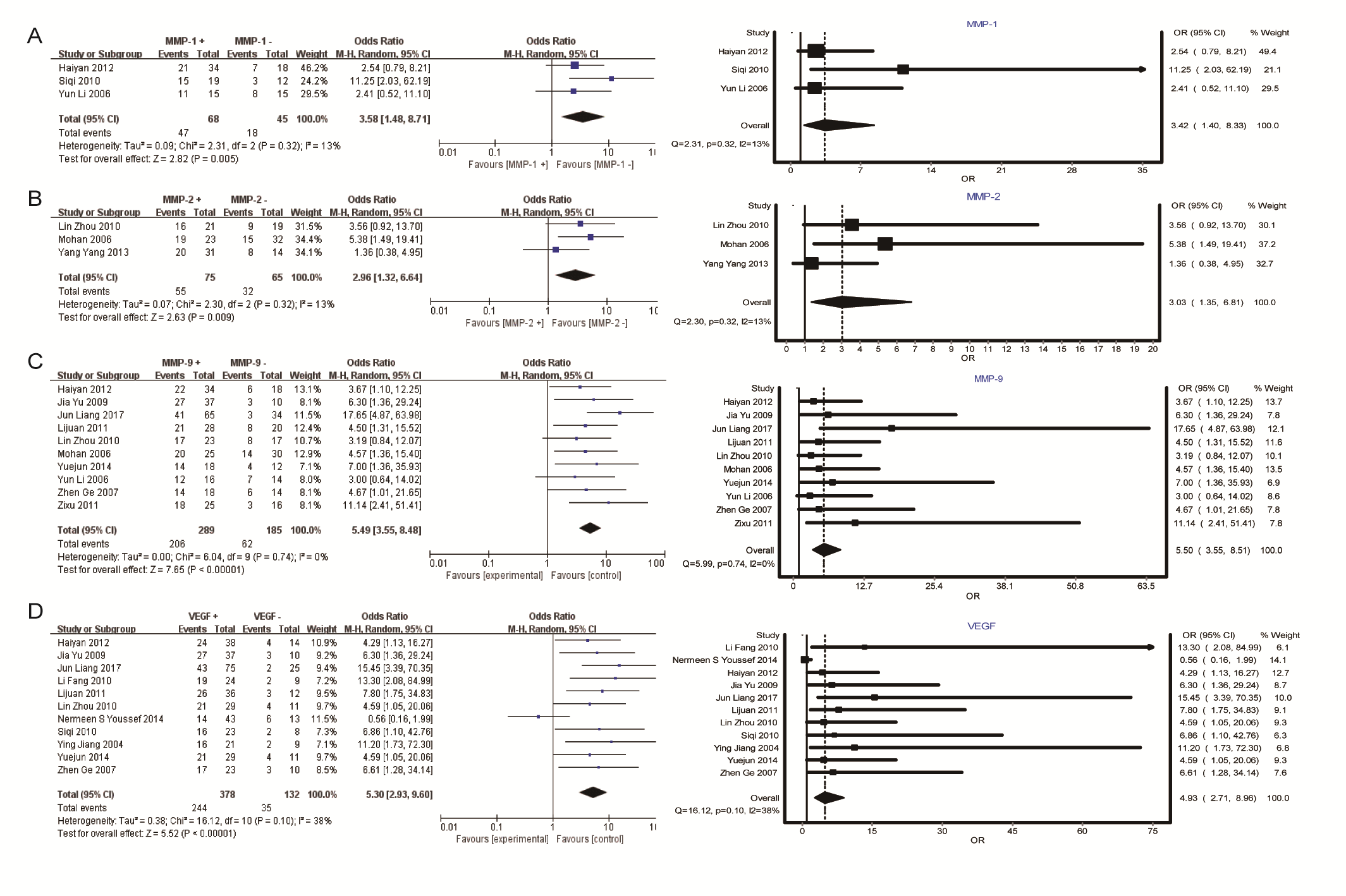

Supplement: Supplementary file 2 — Additional file 2: Figure S2. Association between MMP/VEGF expression and retinoblastoma differentiation. (A) MMP-1. (B) MMP-2. (C) MMP-9. (D) VEGF. The forest plots on the left side show the results of the random-effects model generated using Review Manager. The forest plots on the right side were generated using MetaXL with a quality-effects model. [file 12967_2019_1975_MOESM2_ESM.png]

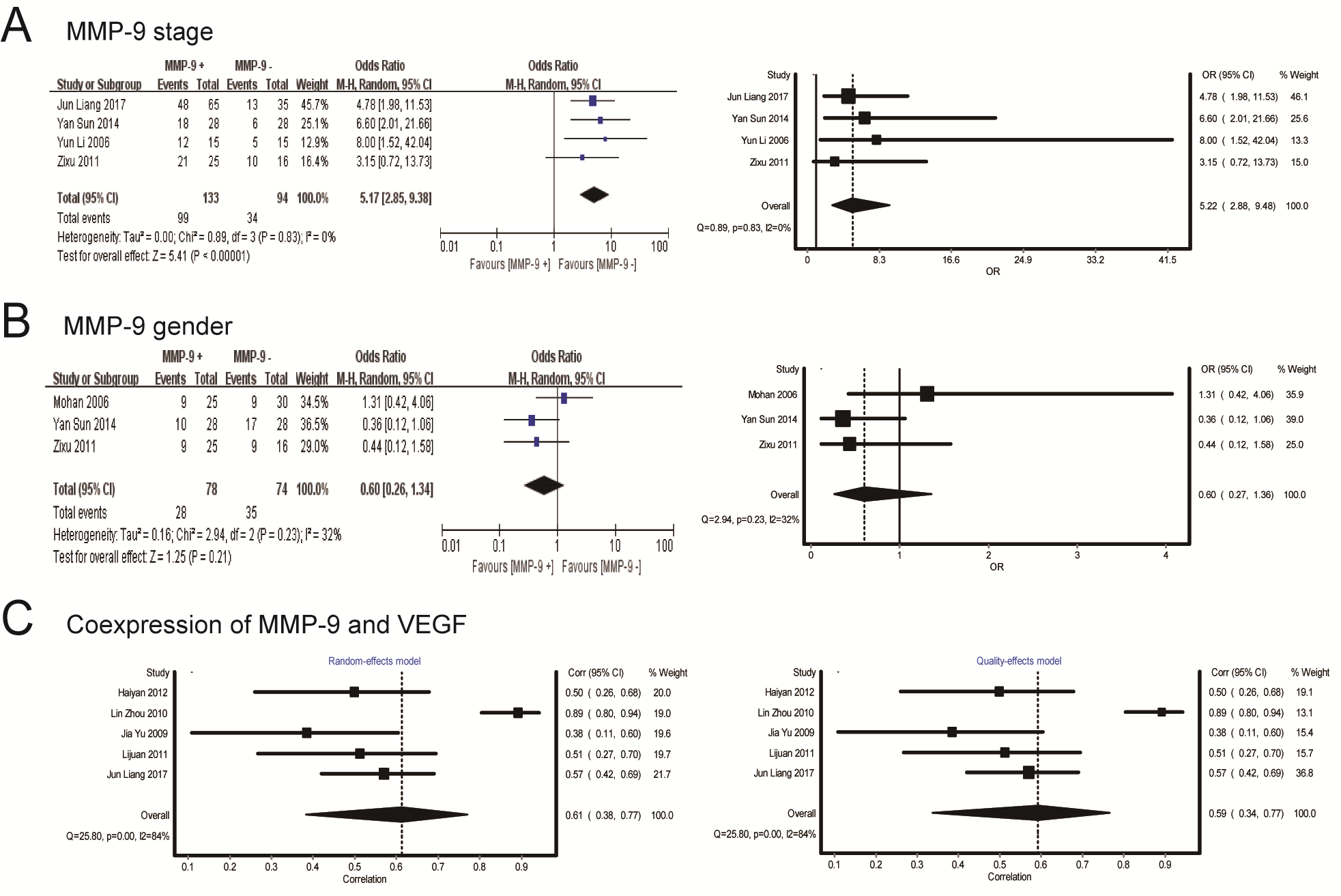

Supplement: Supplementary file 3 — Additional file 3: Figure S3. Association between MMP-9 expression and retinoblastoma clinical stage, patient gender, and VEGF expression. (A) MMP-9 and clinical stage. (B) MMP-9 and patient gender. (C) MMP-9 and VEGF coexpression. The forest plots on the left side show the results of the random-effects model generated using Review Manager. The forest plots on the right side were generated using MetaXL with a quality-effects model. [file 12967_2019_1975_MOESM3_ESM.png]

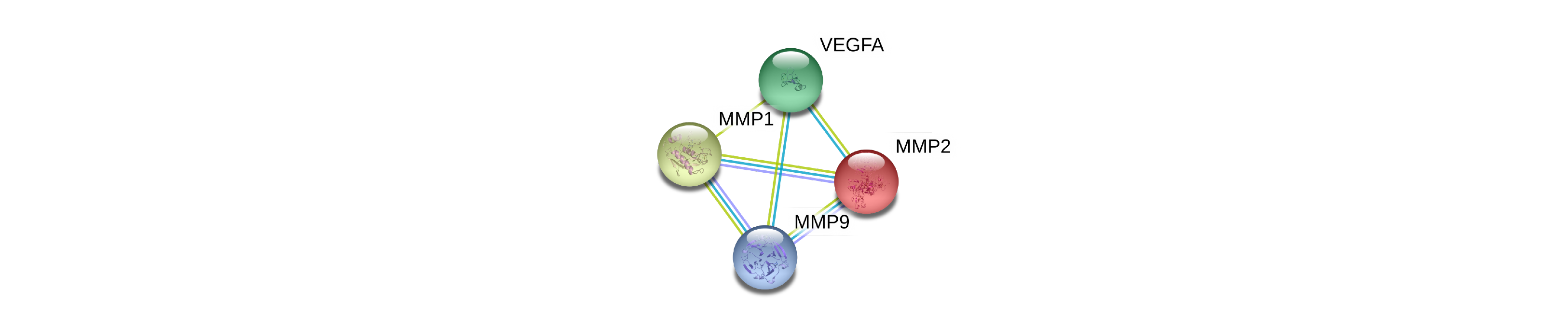

Supplement: Supplementary file 4 — Additional file 4: Figure S4. Protein–protein interaction (PPI) networks between MMP-1, MMP-2, MMP-9 and VEGF. Edges with different colors represent protein–protein associations. Blue edges represent the association from curated databases. Yellow edges have confirmed association by text mining. Purple edges represent the protein homology. [file 12967_2019_1975_MOESM4_ESM.png]

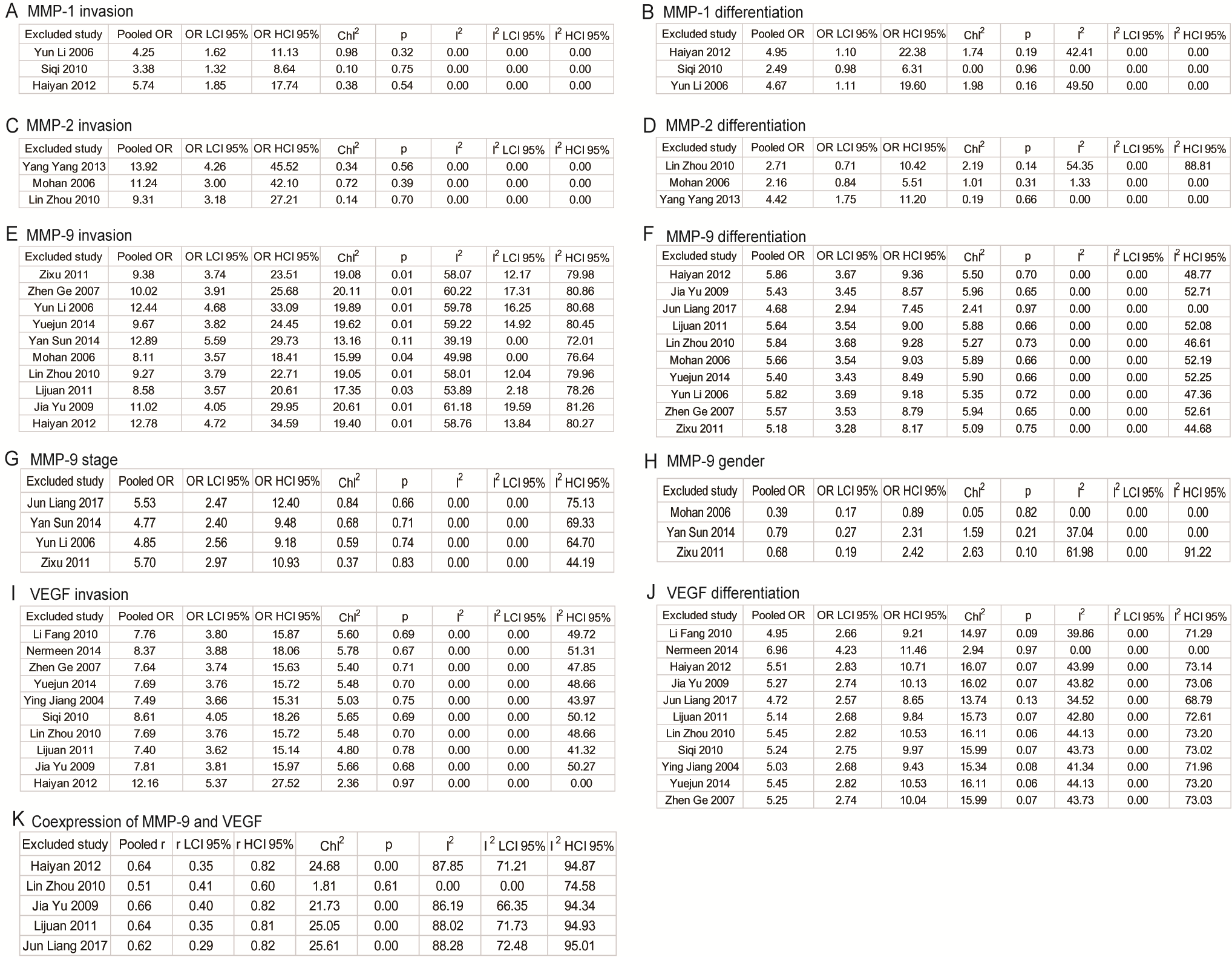

Supplement: Supplementary file 5 — Additional file 5: Figure S5. Sensitivity analysis evaluating the impact of individual studies on the pooled results. (A) MMP-1 and invasion. (B) MMP-1 and differentiation. (C) MMP-2 and invasion. (D) MMP-2 and differentiation. (E) MMP-9 and invasion. (F) MMP-9 and differentiation. (G) MMP-9 and stage. (H) MMP-9 and gender. (I) VEGF and invasion. (J) VEGF and differentiation. (K) MMP-9 and VEGF coexpression. [file 12967_2019_1975_MOESM5_ESM.png]

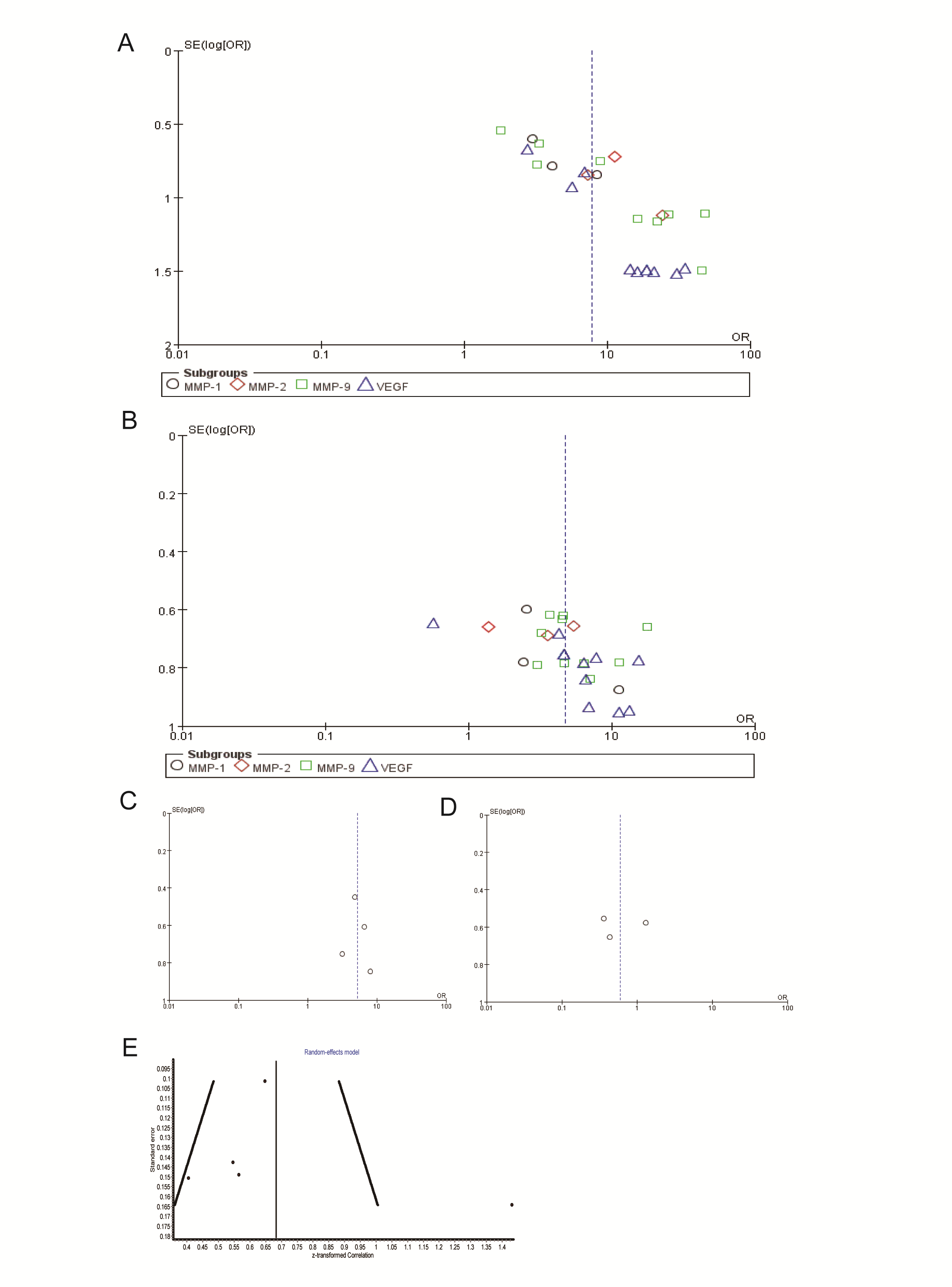

Supplement: Supplementary file 6 — Additional file 6: Figure S6. Funnel plot for publication bias. (A) Tumor invasion. (B) Tumor differentiation. (C) Clinical stage. (D) Gender. (E) Coexpression of MMP-9 and VEGF. [file 12967_2019_1975_MOESM6_ESM.png]
